# Enforced expression of microRNA-21 influences the replication of varicella-zoster virus by triggering signal transducer and activator of transcription 3

**DOI:** 10.3892/etm.2014.1588

**Published:** 2014-02-26

**Authors:** YAN LI, RINA WU, ZHONGRONG LIU, JIANYONG FAN, HUILAN YANG

**Affiliations:** 1 Southern Medical University, Guangzhou, Guangdong 510515, P.R. China; 2 Department of Dermatology, Affiliated Hospital of Inner Mongolia Medical University, Hohehot, Inner Mongolia 010050, P.R. China; 3 Department of Dermatology, General Hospital of Guangzhou Military Command of PLA, Guangzhou, Guangdong 510010, P.R. China

**Keywords:** signal transducer and activator of transcription 3, microRNA-21, varicella-zoster virus

## Abstract

Varicella-zoster virus (VZV) causes chronic pain and serious complications, including zoster paresis. However, the mechanism of VZV replication, a critical part of VZV pathogenesis, remains largely unknown and was investigated in the present study. The upregulation of microRNA-21 (miR-21) was identified following VZV infection *in vitro* by quantitative polymerase chain reaction. The hypothesis that the overexpression of miR-21 activates the signal transducer and activator of transcription 3 (STAT3) signaling pathway was validated by measuring the mRNA expression levels of STAT3 and the anti-apoptotic protein survivin in human malignant melanoma (MeWo) and human embryonic lung fibroblast (HELF) cell lines transfected with miR-21-mimic and comparing them with those in cells transfected with miR-control. To further study the interaction of miR-21, STAT3 and VZV replication, the effects of miR-21 overexpression and STAT3 knockdown were evaluated. Higher virus titers were detected when miR-21 was upregulated *in vitro*. Moreover, it was identified that significantly lower virus titers were present in MeWo cells in which STAT3 was knocked down. In addition, the overexpression of miR-21 did not stimulate VZV replication in the MeWo cell line when the STAT3 gene was silenced. Therefore, the observations of the present study indicate that the enforced expression of miR-21 promotes the replication of VZV by activating STAT3 *in vitro*.

## Introduction

Varicella-zoster virus (VZV) belongs to the α-herpesvirus family and causes varicella (chickenpox) with primary infection and zoster (shingles) during reactivation from a latent state. VZV causes chronic pain or postherpetic neuralgia in patients, but may also cause zoster paresis in the arm, leg, diaphragm or abdominal muscles (1,2). The life cycle of VZV relies on its tropism for T cells, skin and neurons (3–5). VZV replication is an important part of the life cycle; however, the replication mechanisms remain largely unknown.

MicroRNAs (miRNAs) consist of ~22 nucleotides and are small, noncoding and functional RNAs. Due to the numerous functions of miRNAs, they play crucial roles in the pathogenesis of cancers and infections and in particular viral infections (6–8). It has been shown that miRNAs function to activate interferon (IFN)-mediated antiviral activity, suppress the viral counter-responses to cell restriction and affect the viral replication and thus, the pathogenesis of viral infections (7). miRNAs have been documented to have diverse effects in terms of viral replication. Human immunodeficiency virus-1 mRNA is suppressed by a cluster of miRNAs, including miR-28, miR-125b, miR-150, miR-223 and miR-382 (9). Numerous other miRNAs function as inhibiting factors and interfere with viral replication. For example, miR-181 suppresses the respiratory syndrome virus and miR-17-29 suppresses hepatitis B virus (HBV) (10,11). Conversely, miR-501 increases HBV production via the same mechanism by which miR-126 affects coxsackievirus (12,13). Therefore, miRNAs may affect viral replication as suppressors or promoters. However, to date no miRNAs that impact the replication of VZV have been identified.

A major miRNA that plays a crucial role in cancer, cardiovascular diseases, inflammation and virus infection is miRNA-21 (miR-21) (14). It has been shown to affect immunity and viral replication. With respect to viral replication, upregulated miR-21 suppresses hepatitis C virus (HCV)-triggered type I IFN production, which promotes HCV replication. However, dysexpression of miR-21 has been revealed to be an antiviral factor in specific viruses, including Epstein Barr virus (EBV) and HBV (15–17). With regard to immunity, miR-21 is important in maintaining the effector phase of T cells and regulating the Th1 immune response and immune cell functions (14). In addition, miR-21 has been shown to directly target signal transducer and activator of transcription 3 (STAT3) and markedly activate it, subsequently modulating immune functions (18).

STAT3 is one of seven STAT transcription factors that comprehensively affects cell survival, cell cycle progression and homeostasis (19,20). Moreover, STAT3 is important in the pathogenesis of a number of viruses, including γ-herpesviruses, Kaposi’s sarcoma herpesvirus, EBV and herpesvirus saimiri (21,22). A study has found that VZV triggers the STAT3 signaling pathway and activates STAT3, which increases VZV replication via a mechanism dependent on the anti-apoptotic protein survivin (23). Considering the numerous mechanisms and associations among miRNA, virus infection and STAT3, it possible that certain miRNAs may interact with STAT3 and VZV replication.

In the present study, by ectopically increasing miR-21 and knocking down STAT3, the effect of miR-21 expression and STAT3 on the replication of VZV was investigated.

## Materials and methods

### Cell lines and virus

Human malignant melanoma cells (MeWo) and human embryonic lung fibroblasts (HELF) were HELF were purchased from Type Culture Collection of the Chinese Academy of Sciences (Shanghai, China) and grown in Dulbecco’s minimal essential medium (Gibco-BRL, Carlsbad, CA, USA) supplemented with 10% fetal bovine serum in a humidified incubator containing 5% CO_2_ at 37°C. The parental Oka strain of VZV was acquired from Wuhan Institute of Virology, Chinese Academy of Sciences (Wuhan, China). MeWo cells were used to propagate VZV by co-cultivating infected cells with uninfected cells at a ratio of 1/5.

### Cell-free virus preparation and virus infection

Cell-free VZV was provided by Beijing Wantai Biological Pharmacy Enterprise Co., Ltd. (Beijing, China). VZV-infected cells were suspended in a cryoprotective solution (Beijing Wantai Biological Pharmacy Enterprise Co., Ltd.). Cells were lysed by shaking vigorously with 1-mm glass beads. Next, the lysates were centrifuged at 1,240 × g for 10 min at 4°C. The supernatant was stored at −70°C. Viral titers were determined using a standard VZV plaque assay with MeWo cells. The titer of the VZV stock was 7.9×10^6^ plaque forming units (PFU)/ml. The cells were infected with VZV at a multiplicity of infection (MOI) of 1 × 10^−3^.

### RNA/DNA extraction and quantitative polymerase chain reaction (qPCR)

DNA and RNA were extracted from cells using DNeasy and RNeasy kits (Qiagen, Valencia, CA, USA). The expression levels of cellular miR-21 were detected with a *mir*Vana miRNA detection kit (Ambion, Inc., Austin, TX, USA) and qPCR primer sets. U6 small nuclear RNA was used as an internal control. The mRNA expression levels of STAT3 and survivin were assayed using GAPDH as an internal control. The ΔΔCt method was used for relative quantification. The primers were as follows: Survivin, forward, CAT GGG TGC CCC GAC GTT G; and reverse, GCT CCG GCC AGA GGC CTCA A; STAT3, forward, GGG TGG AGA AGG ACA TCA GCG GTA A; and reverse, GCC GAC AAT ACT TTC CGA ATC C; and GAPDH, forward, GGA GTC AAC GGA TTT GGT C; and reverse, GGA ATC ATT GGA ACA TGT AAA C.

### Western blot analysis

Following seeding or transfection, western blotting of the cells were performed by a standard protocol. Briefly, cells were washed with PBS and lysed. Next, the lysates were centrifuged at 13,000 × g at 4°C for 30 min. The supernatants were subjected to 10% SDS-PAGE. Following electrophoresis, proteins were transferred onto nitrocellulose membranes and detected using human monoclonal STAT3 antibodies (R&D Systems, Minneapolis, MN, USA) or β-actin antibodies (Sigma-Aldrich, St. Louis, MO, USA). Secondary antibodies conjugated to horseradish peroxidase were goat anti-mouse IgG or goat anti-rabbit IgG (Pierce Biotechnology, Inc., Rockford, IL, USA). Enhanced chemiluminescent detection systems (SuperSignal West Femto; Pierce Biotechnology, Inc.) were used for detection.

### Gene knockdown and transfection

The expression vector, pSUPERneo, was acquired from OligoEngine (Seattle, WA, USA). The pSUPERSTAT3si plasmid was constructed with a STAT3-specific RNAi sequence (GAT CCC CTT CAG ACC CGT CAA CAA ATT CAA GAG ATT TGT TGA CGG GTC TGA AGT TTT T) which was cloned to the pSUPERneo vector with *Eco*RI/*Hin*dIII (Promega Corporation, Madison, WI, USA). pSUPERSTAT3si was used to conduct the knockdown of the human STAT3 gene (24). STAT3 depletion was validated by western blot analysis. Cells were transfected with an miR-21-mimic or miR-control (Shanghai GenePharma Co., Ltd., Shanghai, China) at a concentration of 100 nM using Lipofectamine 2000 (Invitrogen Life Technologies, Carlsbad, CA, USA).

### Statistical analysis

The data are normalized as mean ± standard deviation. Student’s t-tests were conducted to compare differences in two groups. Comparisons among multiple samples were made by ANOVA. SPSS 17.0 software (SPSS, Inc., Chicago, IL, USA) was utilized to perform the statistical analysis. P<0.05 was considered to indicate a statistically significant result.

## Results

### miR-21 expression increases in HELF and MeWo cell lines following VZV infection

**Figure 1:**
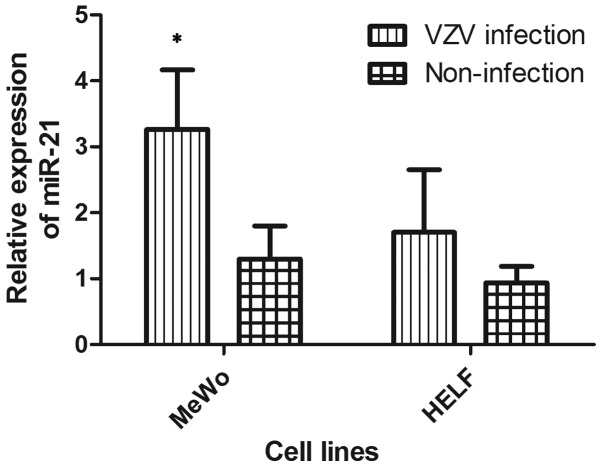
Expression of miR-21 was upregulated in MeWo and HELF cell lines following VZV infection at a MOI of 10^−3^ for 24 h. Expression levels of miR-21 in MeWo and HELF cell lines infected with VZV were assayed by qPCR and compared with those of non-infected cells. The experiments were conducted separately and in triplicate. Data are normally distributed and were statistically analyzed using the Student’s t-test. ^*^P<0.05 compared with non-infected cells of the same cell line. miR-21, microRNA-21; MeWo, human malignant melanoma cells; HELF, human embryonic lung fibroblasts; VZV, varicella-zoster virus; MOI, multiplicity of infection; qPCR, quantitative polymerase chain reaction.

miR-21 has a multifunctional role in virus infection. To demonstrate the effect that miR-21 has on VZV infection, the expression of miR-21 in HELF and MeWo was examined by relative qPCR following VZV infection. The results showed that miR-21 was significantly upregulated in MeWo cells following VZV infection, compared with that in MeWo cells without viral infection (P=0.03). However, the results indicated that the expression level of miR-21 was not significantly higher in VZV-infected HELF cells compared with that in HELF cells without VZV infection. The mean expression level of miR-21 in HELF cells infected with VZV was higher than that without VZV infection (1.7 and 0.93, respectively), as shown in Fig. 1. This indicates that the expression of miR-21 is increased *in vitro* following VZV infection.

### Overexpression of miR-21 is associated with the activation of the STAT3 signaling pathway in vitro

**Figure 2:**
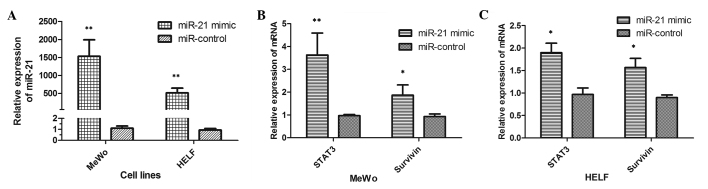
Overexpression of miR-21 is associated with the activation of STAT3 in MeWo and HELF cell lines. (A) Expression of miR-21 was analyzed by qPCR in MeWo and HELF cells transfected with miR-21-mimic at a concentration of 100 nM and compared with the expression level in cells transfected with miR-control. (B) Differences in mRNA expression levels of STAT3 and the anti-apoptotic protein survivin were analyzed between MeWo cells transfected with miR-21-mimic and those transfected with miR-control. (C) mRNA expression levels of STAT3 and survivin were compared between HELF cells transfected with miR-21-mimic and those transfected with miR-control. Data were statistically analyzed using the Student’s t-test. ^*^P<0.05 and ^**^P<0.01 compared with miR-control. miR-21, microRNA-21; STAT3, signal transducer and activator of transcription 3; MeWo, human malignant melanoma cells; HELF, human embryonic lung fibroblasts; qPCR, quantitative polymerase chain reaction.

The ectopic expression of miR-21 was increased by transfection with an miR-21-mimic to investigate whether or not there was an association between miR-21 and STAT3. The expression of miR-21 was confirmed, which certified the efficiency of transfection, as shown in Fig. 1A. The results showed the cells had been successfully transfected with the miR-21-mimic. Next, the differences in the mRNA expression levels of STAT3 and survivin were compared. The mRNA expression levels of STAT3 (P=0.009) and survivin (P=0.026) were significantly enhanced in the MeWo cells transfected with miR-21-mimic (Fig. 2B). Comparable results were obtained for HELF cells (STAT3, P=0.020; survivin, P=0.034), as shown in Fig. 2C. The results showed that the overexpression of miR-21 stimulated the expression of STAT3 and survivin *in vitro*. These observations indicate that the upregulation of miR-21 is associated with activation of the STAT3 signaling pathway *in vitro*.

### Overexpression of miR-21 promotes VZV replication in vitro

**Figure 3:**
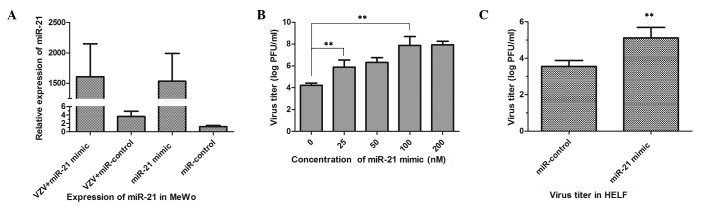
Upregulation of miR-21 stimulates VZV replication. (A) Expression of miR-21 in MeWo cells transfected with 100 nM miR-control or 100 nM miR-21-mimic, with and without VZV infection at a MOI of 1 × 10^−3^ for 24 h, to investigate the interaction of VZV and upstream miR-21. (B) Virus titers, performed by standard VZV plaque assays, were compared among MeWo cells transfected with miR-21-mimic at concentrations of 0, 25, 50, 100 and 200 nM following VZV infection for 24 h. (C) Comparison between the virus titers in HELF cells transfected with 100 nM miR-21-mimic and 100 nM miR-control following VZV infection for 24 h. ^*^P<0.05 and ^**^P<0.01 compared with control. miR-21, microRNA-21; MeWo, human malignant melanoma cells; HELF, human embryonic lung fibroblasts; VZV, varicella-zoster virus; MOI, multiplicity of infection.

Since miR-21 overexpression was identified in MeWo cell lines, whether changes in miR-21 expression were associated with changes in VZV replication was investigated. Transfection of MeWo cells with miR-21-mimic or miR-control was performed in order to detect any differences in replication following VZV infection at a MOI of 1 × 10^−3^. Detection of miR-21 expression confirmed the efficiency of transfection, as shown in Fig. 3A. Furthermore, the results indicated that the higher the expression levels of miR-21, the higher the virus titer was. However, the virus titer did not increase significantly when miR-21 was transfected at a concentration of 200 nM, as shown in Fig. 3B. The virus titer in HELF cells transfected with 100 nM miR-21-mimic was also detected. The results showed the mean virus titer to be 1.29×10^6^ PFU/ml in HELF cells transfected with miR-21-mimic, compared with 3.55×10^3^ PFU/ml in cells transfected with the miR-control (Fig. 3C). Therefore, this indicates that the ectopic expression of miR-21 contributes to VZV replication.

### Overexpression of miR-21 promotes VZV replication by STAT3 activation in vitro

**Figure 4:**
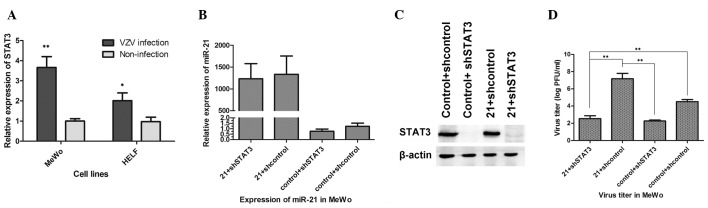
Overexpression of miR-21 promotes VZV replication by activating STAT3. (A) Relative mRNA expression levels of STAT3 in MeWo and HELF cells following VZV infection and non-infection. (B) Expression levels of miR-21 in the 21 + shSTAT3, 21 + shcontrol, control + shSTAT3 and control + shcontrol groups in MeWo cells infected with VZV for 24 h. Groups were assigned according to transfection with miR-21 (21) or control, and knockdown of STAT3 (shSTAT3) or control knockdown (shcontrol). (C) Expression of miR-21 and the silencing of the STAT3 gene was validated by western blot analysis of the four groups. (D) Virus titers were compared among the four groups. ^*^P<0.05 and ^**^P<0.01. miR-21, microRNA-21; STAT3, signal transducer and activator of transcription 3; MeWo, human malignant melanoma cells; HELF, human embryonic lung fibroblasts; VZV, varicella-zoster virus.

The relative mRNA expression levels of STAT3 were assayed following VZV infection to confirm the association between VZV infection and the STAT3 signaling pathway. The results indicated that the relative mRNA expression level of STAT3 in MeWo cells infected with VZV was significantly higher compared with that in MeWo cells not infected with VZV (P=0.001). Comparable results were obtained in HELF cells (P=0.016; Fig. 4A).

To further investigate the associations among miR-21, STAT3 and VZV infection, the expression levels of miR-21 were increased by transfection with miR-21-mimic and STAT3 genes were silenced following VZV infection in MeWo cells. The four groups were designated as follows: 21 + shSTAT3, cells co-transfected with miR-21-mimic and STAT3 knockdown; 21 + shcontrol, cells co-transfected with miR-21-mimic and control knockdown; control + shSTAT3, cells co-transfected with miR-control and STAT3 knockdown; and control + shcontrol, cells co-transfected with miR-control and control knockdown. The expression of miR-21 was confirmed in the in MeWo cells of the four groups. Higher miR-21 expression levels were observed in the 21 + shSTAT3 and 21 + shcontrol groups compared with those in the other two groups, as shown in Fig. 4B. The expression of STAT3 protein was detected by western blot analysis. The results showed that the expression level of STAT3 protein was extremely low in the 21 + shSTAT3 and control + shSTAT3 groups, as shown in Fig. 4C. This demonstrated that the STAT3 gene was knocked down effectively. Finally, the virus titers were assayed and the mean titers were 3.47×10^2^ PFU/ml (21 + shSTAT3), 1.48×10^7^ PFU/ml (21 + shcontrol), 1.82×10^2^ PFU/ml (control + shSTAT3) and 3.23×10^4^ PFU/ml (control + shcontrol), as shown in Fig. 4D. These results indicate that VZV replication was significantly decreased with STAT3 knockdown. In addition, the overexpression of miR-21 stimulated VZV replication, but not when the STAT3 gene was silenced in MeWo cell lines. The results indicate that the ectopic overexpression of miR-21 increased VZV replication by activating STAT3 *in vitro.*

## Discussion

MiRNAs, including miR-21, have important effects on viral replication, and function as inhibiting or enhancing factors (7). Moreover, it has been documented that miR-21 may be highly involved in certain cell-signaling pathways that modulate the immune system, including the IFN, nuclear factor κB (NF-κB), extracellular signal-regulated kinases-mitogen-activated protein kinase and STAT3 signaling pathways (14,25). It has been hypothesized that associations exist among miRNA, the immune system and viral replication. Sen *et al* revealed that STAT3 promoted VZV replication (23). However, it remains unknown as to whether miRNAs regulate the mechanism of VZV replication.

In the present study, the expression of miR-21 was observed to be upregulated significantly in MeWo cells following infection with VZV. Upregulation of miR-21 also occurred in HELF cells following VZV infection; however, the increase in miR21 levels in HELF cells was not found to be significant. To confirm the effect of miR-21 on VZV replication, MeWo and HELF cell lines were transfected with miR-21-mimic. The transfection was shown to stimulate VZV replication, which indicates that miR-21 plays an important role as a promoter in VZV replication.

STAT3 responds to a variety of signals, including growth factors, cytokines and oncogenes. STAT3 is modulated by the miR-17-92 cluster, which affects tumorigenesis (26,27). Furthermore, the miR-21/STAT3 interaction has been studied extensively. Expression of miR-21 and upstream STAT3 occurs simultaneously in myeloma cells and the knockdown of STAT3 prohibits the upregulation of miR-21 (28). However, conflicting results have been produced with regard to the miR-21/STAT3 interaction in human glioma cells. In one study, it was found that increased STAT3 expression resulted in the downregulation of miR-21. This was confirmed by a further study that evaluated changes in the expression of miR-21 with the overexpression or knockdown of STAT3 (29). The conflicting results indicate that the miR-21/STAT3 interaction may be largely determined by the microenvironment.

In the present study, miR-21 was overexpressed in MeWo and HELF cell lines by transfection with miR-21-mimic. The upregulation of STAT3 occurred concurrently with miR-21 overexpression in these cell lines. This was further supported by evaluating miR-21 expression in cells with silenced STAT3 genes, as shown in Fig. 4B. The observations demonstrated that the miR-21/STAT3 interaction was reinforced mutually in MeWo and HELF cell lines. In addition, previous observations indicated that the upregulation of miR-21 had positive effects on VZV replication. Subsequently, the association between the STAT3/miR-21 interaction and its effects on viral replication and evasion of the host immune system required investigation. Specifically, the roles that miR-21/STAT3 interactions play in VZV replication were considered. When miR-21 was upregulated, the viral titer when STAT3 was knocked down was found to be significantly lower compared with the viral titer for non-silenced STAT3. This result indicated that the miR-21/STAT3 interaction plays a positive role in VZV replication. Additionally, when STAT3 was knocked down, a comparison of the viral titers in cells transfected with miR-21-mimic with those in cells transfected with miR-control indicated that the mechanism by which miR-21 promotes VZV replication is strictly regulated by STAT3, .

Several viruses have been shown to directly or indirectly decrease the expression of factors associated with the host innate immune system, including proteins, genes and cell signaling pathways, in order to enhance their own replication (30). VZV replicates not only by preventing IFN induction via inhibition of the IFN regulatory factor 3 mediated IFN-β pathway, but also by inhibiting certain signaling pathways, including NF-κB and STAT1 pathways (31–33). Conversely, host cellular factors may also be used to viral advantage. The activation of STAT3 and upregulation of survivin is a necessary mechanism of VZV pathogenesis and is important for the pathogenesis of lytic and tumorigenic herpesviruses. The present study of VZV replication further supports the hypothesis that viruses use cellular factors to their advantage.

In conclusion, cellular miR-21 was found to be a promoting factor of VZV replication. The study also demonstrated that STAT3 was involved in the mechanism by which miR-21 modulates VZV replication. In addition, the enforced expression of miR-21 was shown to promote the replication of VZV by activating STAT3 *in vitro*. This study increased the understanding of the mechanism of VZV replication, a critical part of the VZV pathogenesis.
